# Identification of hub genes significantly linked to tuberous sclerosis related-epilepsy and lipid metabolism via bioinformatics analysis

**DOI:** 10.3389/fneur.2024.1354062

**Published:** 2024-02-14

**Authors:** Wang Weiliang, Ren Yinghao, Hou Weiliang, Zhang Xiaobin, Yang Chenglong, An Weimiao, Xu Fei, Wang Fengpeng

**Affiliations:** ^1^Epilepsy Center, Xiamen Humanity Hospital Fujian Medical University, Xiamen, Fujian, China; ^2^Department of Dermatology, Xiamen Humanity Hospital Fujian Medical University, Xiamen, Fujian, China; ^3^Department of Neurosurgery, Huashan Hospital, Shanghai Medical College, State Key Laboratory of Medical Neurobiology, Ministry of Education Frontiers Center for Brain Science and Institutes of Brain Science, Fudan University, Shanghai, China; ^4^Department of Neurosurgery, The Cancer Hospital of Harbin Medical University, Harbin, Heilongjiang, China; ^5^Department of Pharmacogenomics, College of Bioinformatics Science and Technology, Harbin Medical University, Harbin, China

**Keywords:** tuberou sclerosis complex, epilepsy, lipid metabolism, bioinformatics analysis, biomarkers

## Abstract

**Background:**

Tuberous sclerosis complex (TSC) is one of the most common genetic causes of epilepsy. Identifying differentially expressed lipid metabolism related genes (DELMRGs) is crucial for guiding treatment decisions.

**Methods:**

We acquired tuberous sclerosis related epilepsy (TSE) datasets, GSE16969 and GSE62019. Differential expression analysis identified 1,421 differentially expressed genes (DEGs). Intersecting these with lipid metabolism related genes (LMRGs) yielded 103 DELMRGs. DELMRGs underwent enrichment analyses, biomarker selection, disease classification modeling, immune infiltration analysis, weighted gene co-expression network analysis (WGCNA) and AUCell analysis.

**Results:**

In TSE datasets, 103 DELMRGs were identified. Four diagnostic biomarkers (ALOX12B, CBS, CPT1C, and DAGLB) showed high accuracy for epilepsy diagnosis, with an AUC value of 0.9592. Significant differences (*p* < 0.05) in Plasma cells, T cells regulatory (Tregs), and Macrophages M2 were observed between diagnostic groups. Microglia cells were highly correlated with lipid metabolism functions.

**Conclusions:**

Our research unveiled potential DELMRGs (ALOX12B, CBS, CPT1C and DAGLB) in TSE, which may provide new ideas for studying the psathogenesis of epilepsy.

## Introduction

Tuberous sclerosis complex (TSC) is a rare autosomal dominant genetic disorder characterized by the growth of benign tumors in multiple organ systems, including the skin, kidneys, lungs, heart, and brain. A common feature of TSC is epilepsy (1). Epileptic seizures are a progressively worsening and dynamic process in which several cellular, molecular and pathophysiological mechanisms may be involved, including mammalian target of rapamycin (mTOR) dysregulation and synaptic abnormalities (2). TSC is a neurodevelopmental disorder caused by mutations in the *TSC1* or *TSC2* genes (3). The proteins encoded by these genes are responsible for regulating the signal of the mTOR complex (4). mTOR is part of a complex signal network and plays a crucial role in regulating various cellular processes, including cell growth and metabolism (5).

Most TSC-related manifestations are the result of over-activation of the mammalian target of rapamycin (mTOR) complex. Rapamycin has been widely used in different animal models of TSC-associated epilepsy and has been shown to have antiepileptic potential as it not only inhibits seizures but also prevents seizure development (6). The mTOR pathway has been established to be closely associated with lipid metabolism functions (7). Additionally, the ketogenic diet has shown efficacy in alleviating TSC-associated seizures, and decanoic acid has been found to reduce mTORC1 activity in a model of tuberous sclerosis, including astrocytes derived from TSC patients (8). The cumulative evidence suggests a close association between lipid metabolism and the occurrence of TSE.

Recent studies have shown that metabolism is critical in regulating homeostasis, dormancy and differentiation of neural stem cells (9). Neural stem cells can utilize free fatty acid oxidation to generate energy (10). Under energy-deficient stress conditions, in TSC-deficient cells, high activation of mTORC1 reconfigures metabolism, leading to increased aerobic glycolysis and increased fatty acid synthesis. TSC-deficient cells require autophagy to maintain high mTORC1 activation, possibly through lipid autophagy, to provide lipids as an alternative energy source for oxidative phosphorylation. *In vivo* inhibition of lipid autophagy or its downstream catabolic pathways reversed the defective phenotype induced by TSC1-deficient neural stem cells and reduced tumorigenesis in a mouse model (7). This evidence suggests an important role for the mTOR pathway in influencing lipid metabolism in TSC patients.

The influence of lipid metabolism on epilepsy is likely due to its function as a “secondary fuel” for the brain. Multiple studies have revealed potential impairments in glucose metabolism within regions of the brain affected by epilepsy. Maintaining normal brain function relies heavily on energy, and deficits in energy may disrupt the ionic gradient, leading to neuronal depolarization and epilepsy (5). Ketogenic diets offer ketone bodies like acetoacetic acid and beta-hydroxybutyric acid, acting as alternative energy sources for the brain. Around 50% of individuals, including both children and adults with specific types of epilepsy who can tolerate and adhere to these dietary regimens, experience a decrease in the frequency of seizures. Recent data suggests that incorporating medium-chain triglycerides, which provide caprylic and capric acid—two medium-chain fatty acids—along with ketone bodies as supplementary energy for the brain, proves beneficial in rodent epilepsy models, canines, and human patients with epilepsy (11).

To identify genes closely associated with lipid metabolism and TSE disease progression, we identified differentially expressed lipid metabolism related genes for possible therapeutic targets.

## Materials and methods

### Data source

**Figure 1:**
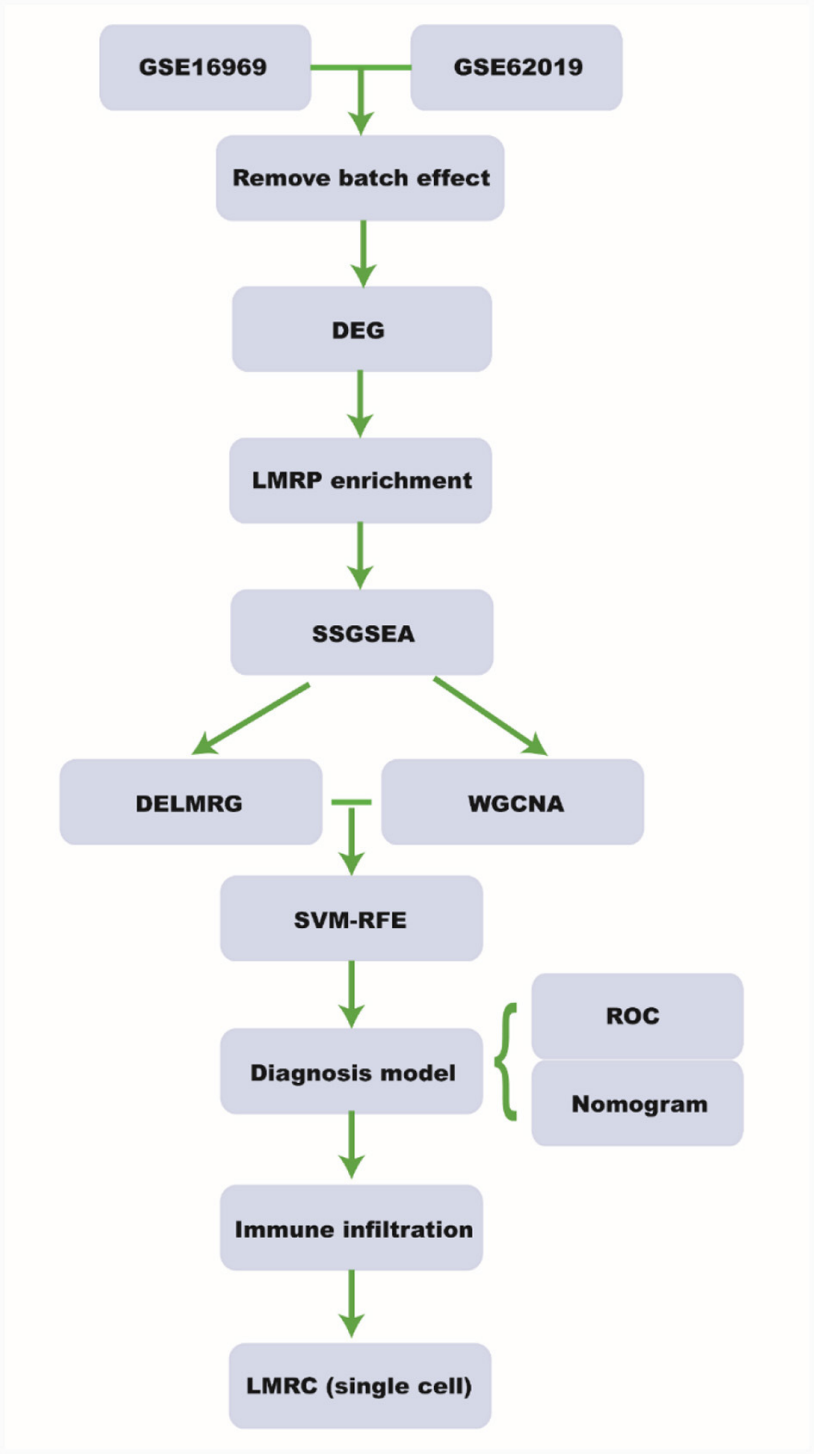
Workflow. DEG, differential expressed gene; LMRP, lipid metabolism related pathway; ssGSEA, single-sample gene set enrichment analysis; DELMRG, differential expressed lipid metabolism related gene; WGCNA, weighted relation network analysis; SVM-RFE, support vector machine-recursive feature elimination; ROC, receiver operating curve; LMRC, lipid metabolism related cell.

Data related to Tuberous Sclerosis and Epilepsy were obtained from the Gene Expression Omnibus (GEO) database (12). Specifically, we downloaded the datasets GSE16969 (13) and GSE62019 (14), as well as single cell sequencing data from GSE201048 (15) (Supplementary Table 1). Lipid metabolism related pathways (LMRPs) were also sourced from the following PubMed articles: PMID35222371 (16), PMID36091041 (17), PMID36860853 (18), and PMID37469520 (19) (Supplementary Table 2). The design and workflow of this study are shown in Figure 1.

### Differentially expressed genes in tuberous sclerosis related epilepsy

GSE16969 and GSE62019 were processed using the “sva” package for acquiring an integrated GEO dataset consisting of 14 samples, among which there were seven tuberous sclerosis related epilepsy (TSE) samples and seven control (CTRL) samples (20). The dataset underwent Principal Component Analysis (PCA). Subsequently, the “limma” package was employed to determine the differentially expressed genes (DEGs) among the various subgroups (TSE vs. CTRL) at |log fold change (FC)| > 0.58 and adjusted *p* < 0.05 (21).

### Gene set enrichment analysis

Gene sets were obtained from the MSigDB database, including the following: “c5.go.v2023.1.Hs.symbols.gmt,” “c2.cp.kegg.v2023.1.Hs.symbols.gmt,” “c2.cp.reactome.v2023.1.Hs. sym bol.gmt,” “c2.cp.wikipathways.v20 23.1.Hs.symbols.gmt,” and “h.all.v2023.1.Hs.symbols.gmt” (22). The resulting dataset underwent enrichment analysis utilizing the GSEA method provided by the “clusterProfiler” package, with adjusted *p* < 0.05 (23). By combining the LMRPs downloaded from the literature, with keywords such as “lipid,” “prostanoid,” “fatty acid,” “cholesterol,” “phosphatidylcholine,” and other metabolism-related keywords, we identified LMRPs that exhibited differential enrichment between the TSE and control groups (24).

### Single sample gene set enrichment analysis

Gene sets for LMRPs with inter-group differences, based on enrichment, underwent ssGSEA analysis in the integrated dataset related to TSE by comparing TSE and control groups. Enrichment scores for each sample, which indicate the activity levels of these pathways, were calculated using the “ssGSEA” algorithm from the R package (25). Pathways activity variances were evaluated between the TSE and control groups through the “lmFit” analysis (21).

### Differentially expressed lipid metabolism related genes

The lipid metabolism related genes (LMRGs) were acquired from the MSigDB database. To identify the DELMRGs, they were intersected with the DEGs. The resulting overlap was illustrated in a Venn diagram. Afterward, we conducted protein-protein interaction (PPI) network analysis on the resulting genes using the STRING database (26). We employed the Cytoscape (27) plugin “cytoHubba” (28) and the Maximal Clique Centrality (MCC) algorithm to pinpoint the ten most pivotal genes within the network based on their MCC scores.

### Weighted gene co-expression network analysis

Hierarchical clustering was conducted using ssGSEA enrichment scores for LMRPs that were associated with inter-group differences. To determine the optimal number of clusters, the “fviz_nbclust” function of the R package “factoextra” was utilized. Clustering results were obtained for samples in the integrated dataset based on their lipid metabolism functions. Additionally, WGCNA was performed on the combined dataset related to TSE (28). In this investigation, WGCNA utilized the amalgamated dataset for TSE as an input to evaluate the connection between the progression of the disease phenotype and various gene modules. In addition, it documented the genes within each module, considering them as feature genes that are specific to the module.

### Risk model construction

The “ggvenn” package was used to generate a Venn diagram by taking the intersection of DELMRGs and lipid metabolism-related module genes identified via WGCNA. The support vector machine-recursive feature elimination (SVM-RFE) algorithm was utilized for the feature selection of LMRGs linked with TSE progression, using the chosen genes (29). Following the selection of feature genes, logistic regression was employed to develop a diagnostic model. Subsequently, a risk diagnostic score was determined according to the gene expression levels and coefficients obtained from multiple regression analysis.


$$\begin{array}{l} \text{ Diagnosis }Score\;=\\ \underset{i}{\sum }Coefficient\;\left({feature\;gene}_{i}\right)*mRNA\;Expression\;\left(feature\;gene_{i}\right) \end{array}$$

The following formula was used to calculate the diagnosis score: A higher AUC (area under the curve) value indicates better diagnostic performance. The receiver operating characteristic (ROC) curve for the TSE progression status risk model was plotted using the “pROC” package (30). After SVM-RFE feature selection and model building, a nomogram (24) was created with the “rms” package.

### Immune infiltration analysis

The expression profile dataset of TSE was uploaded onto the CIBERSORTx website (27). Samples with immune cell enrichment scores greater than zero were selected via data filtration. Later, the specific outcomes of the immune cell infiltration abundance matrix were retrieved and displayed. The distribution of immune cells in high and low diagnostic score sample groups from the TSE dataset were presented using bar plots and box plots. The correlation between immune cells in the groups with high and low diagnostic scores and TSE risk model genes linked to lipid metabolism were computed via Spearman rank correlation analysis. A correlation heat map was produced utilizing the “ggplot2” package.

### Gene set variation analysis

To acquire the reference gene set “h.all.v7.4.symbols.gmt” from the MSigDB database and execute GSVA on an integrated GEO dataset comprising varied groups (high vs. low diagnostic score group) (25). GSVA converts the expression matrix into a pathway enrichment score matrix. We employed the “lmFit” analysis to identify the variations in pathways between the high and low diagnostic score groups (21). After that, we established the Pearson correlation between the feature genes of the diagnostic model and the distinctively regulated pathways of the high and low diagnostic score groups. Visualizations were created in the form of a bubble chart using the “ggcorrplot” package and scatter charts using “ggpmisc” and “ggExtra”.

### AUCell analysis

We conducted an efficient data processing and visualization of the GSE201048 single cell dataset utilizing the “Seurat” package (31). Following this, we employed t-distributed stochastic neighbor embedding (tSNE) to illustrate the subpopulation annotations of the cells. To investigate the functional disparities of lipid metabolism-related cells (LMRCs) among diverse cellular subpopulations, we utilized the “AUCell” package (32) to determine the pathway activity of individual cells based on the single cell expression profiles of GSE201048. We then identified cell clusters with active “gene sets” within the single cell data. Lastly, we scored each cell based on the feature genes of the diagnostic model and gene expression ranking information. The AUC score somewhat indicated the ratio of top-performing genes found in a selection of pathway genes in every cell, signifying the action level of specific gene sets in each cell.

### Gene ontology enrichment analysis

Based on the single-cell expression profiles from GSE201048, we employed the “FindMarkers” function from the “Seurat” package to detect DEGs among various cell subpopulations (31). For identifying the DEGs within the single cell subpopulations, genes satisfying the criteria of |logFC| < = 0.25 and adjusted *p* < 0.05 were selected. Large-scale functional enrichment studies of genes in various dimensions and hierarchical levels were conducted through the widely accepted approach of GO enrichment analysis (33). The analysis was conducted across three dimensions: Biological Process (BP), Molecular Function (MF), and Cellular Component (CC) (34). To identify significantly enriched biological processes and pathways, we utilized the “clusterProfiler” package for GO enrichment analysis (23). The visual representation of the enrichment results was created with the “ggplot2” package (24).

### Statistical analysis

We conducted all data calculations and statistical analyses using R (version 4.2.3). The Benjamini-Hochberg method was applied for multiple testing adjustments. Independent Student's *t*-tests assessed statistical significance for normally distributed variables. For non-normally distributed variables, we used the Wilcoxon test. Spearman's correlation analysis calculated correlation coefficients between different molecules. All *p*-values were two-tailed, and statistical significance was set at *p* < 0.05.

## Results

### Differential expression analysis of TSE data

**Figure 2:**
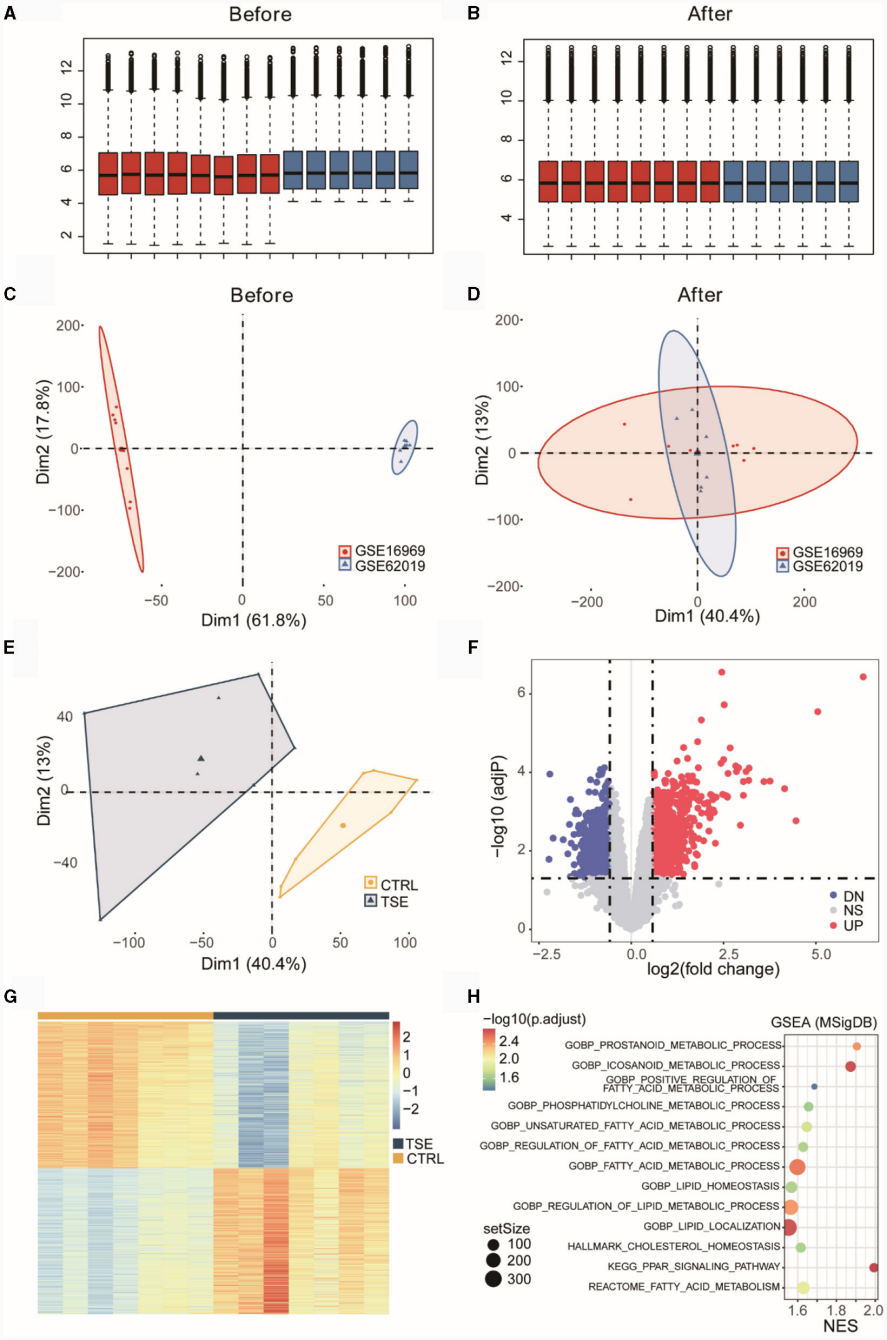
Differential expression analysis of an integrated dataset of TSE. Box plots before **(A)** and after **(B)** batch correction of the merged TSE dataset. Inter-batch analysis using PCA before **(C)** and after **(D)** batch correction of the merged TSE dataset. Differential analysis of the TSE group and the control group using PCA after batch correction of the merged TSE dataset **(E)**. Volcano plot **(F)** and heatmap **(G)** illustrating DEGs between the TSE group and the control group. GSEA (TSE vs. CTRL) enrichment analysis bubble chart **(H)**. NES, normalized enrichment score.

GSE16969 and GSE62019 underwent batch correction and were merged. Box plots before and after batch correction of the combined epilepsy dataset were presented in Figures 2A, B, while Figures 2C, D illustrated the results of PCA for the combined epilepsy dataset, before and after batch correction, respectively. In Figure 2E, certain differences at the transcriptome level between the TSE group and the control group were shown. The differential expression analysis yielded 1,421 DEGs, comprising 708 upregulated genes and 713 downregulated genes, which were graphically displayed as a volcano plot in Figure 2F and a heatmap in Figure 2G. GSEA was performed on pathways from the MSigDB database, specifically GO, KEGG, HALLMARK, REACTOME, and WIKIPATHWAY (Supplementary Table 3). Figure 2H showed that the TSE group was linked to 13 LMRPs, including “GOBP-PROSTANOID-METABOLIC-PROCESS,” “GOBP-ICOSANOID-METABOLIC-PROCESS,” “GOBP-POSITIVE-REGULATION-OF-FATTY-ACID-METABOLIC-PROCESS,” and “GOBP-PHOSPHATIDYLCHOLINE-METABOLIC-PROCESS.”

**Figure 3:**
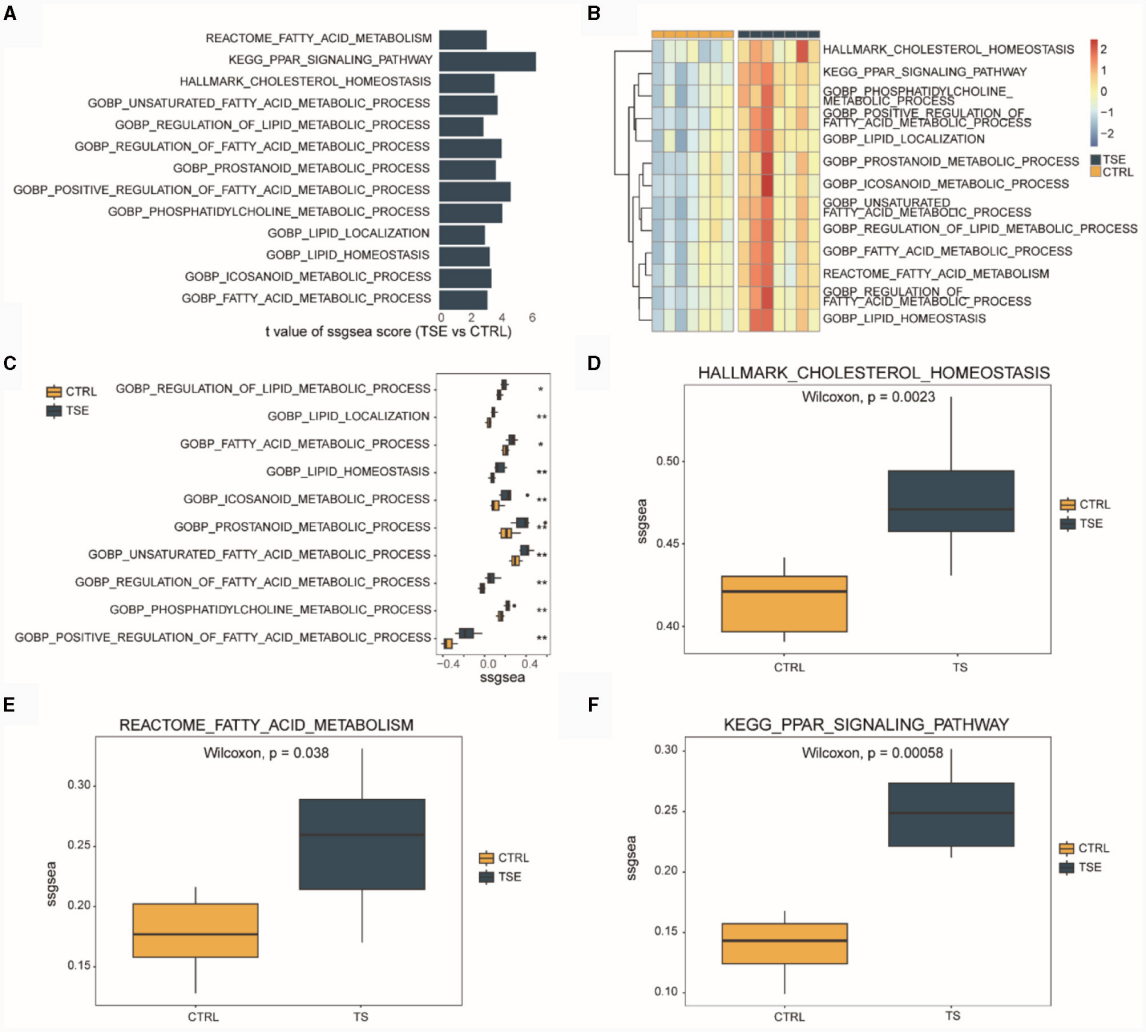
ssGSEA of LMRPs in the integrated dataset related to TSE. **(A)** Differential analysis of LMRPs in the TSE integrated dataset. **(B)** Heatmap of ssGSEA scores for LMRPs in the TSE integrated dataset. Box plots of ssGSEA scores for lipid metabolism-related GO pathways **(C)**, HALLMARK pathways **(D)**, REACTOME pathways **(E)**, and KEGG pathways **(F)** between the TSE group and the control group.

We conducted ssGSEA based on the integrated TSE dataset. Figure 3A displayed LMRPs with differential ssGSEA scores between groups (TSE vs. CTRL). Figure 3B presented ssGSEA scores for 13 LMRPs. Figure 3C shows differential ssGSEA scores for 10 LMRPs related to GO. Figure 3D illustrated differential ssGSEA scores for one pathway related to HALLMARK. Figure 3E displays differential ssGSEA scores for one pathway related to REACTOME, and Figure 3F showed differential ssGSEA scores for one pathway related to KEGG. These results demonstrated that the 13 LMRPs enriched through differential expression analysis also exhibit inter-group differences in ssGSEA scores.

**Figure 4:**
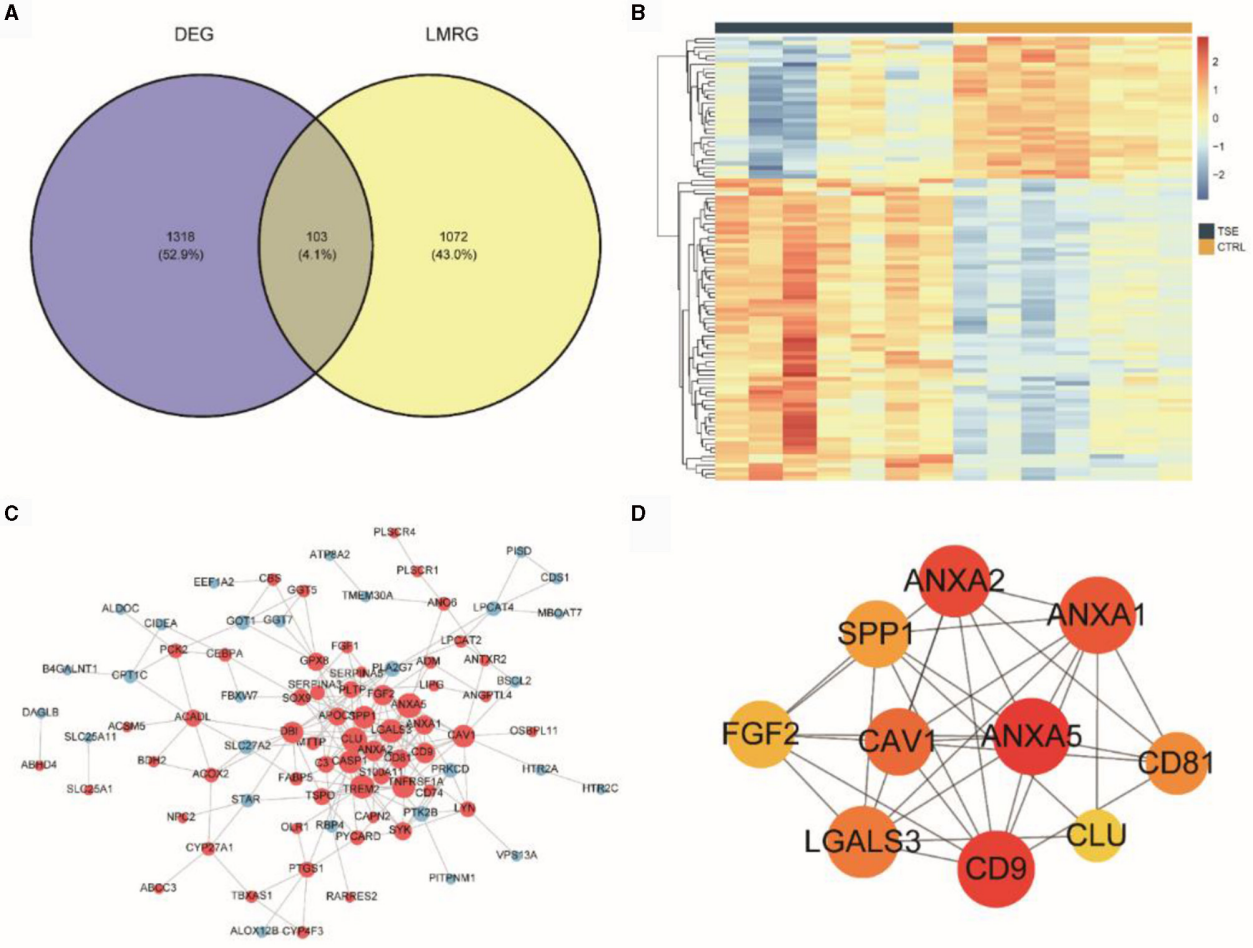
DELMRGs analysis. **(A)** Venn diagram showing the intersection of DEG (TSE vs. CTRL) and LMRG. **(B)** Heatmap depicting the expression of DELMRGs in the TSE and control groups. **(C)** PPI network of DELMRGs. **(D)** Top 10 hub genes based on MCC scores.

We intersected 13 LMRGs (1,175 unique genes after deduplication) with the DEGs between the TSE and control groups, resulting in 103 DELMRGs (Figure 4A). Subsequently, we examined the expression patterns of these genes in the TSE and control groups (Figure 4B). The results showed that 33 genes were downregulated in the TSE group, while 70 genes were upregulated in the TSE group. We constructed a protein-protein interaction (PPI) network (Figure 4C). Figure 4D represented the top 10 hub genes in the PPI network based on MCC scores, with ANXA5 having the highest MCC score and degree.

### Results of WGCNA

**Figure 5:**
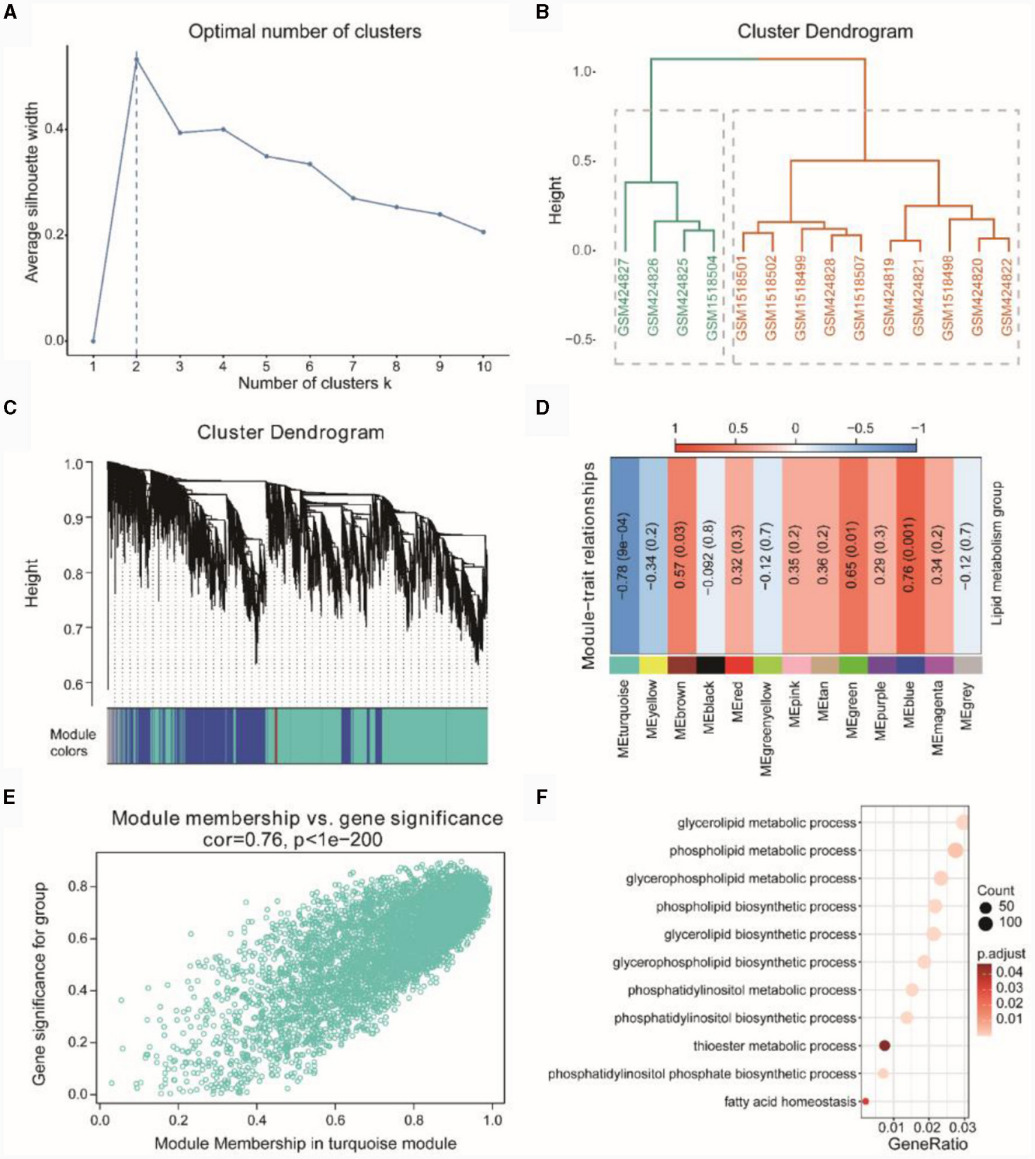
WGCNA of TSE. **(A)** Optimal number of clusters line graph for hierarchical clustering. **(B)** Hierarchical clustering result dendrogram. **(C)** Gene clustering dendrogram based on topological overlap (above) and module color assignments for different gene clusters (below). **(D)** Heatmap showing the correlation between modules and phenotypic traits. **(E)** Scatterplot of Gene Significant (GS) and Module Membership (MM) in the turquoise module. **(F)** Gene enrichment entries related to lipid metabolism in the turquoise module.

Figure 5A indicated the optimal number of clusters obtained through hierarchical clustering, with the result showing that the optimal number of clusters was 2. In Figure 5B, the hierarchical clustering results demonstrated that GSM424827, GSM424826, GSM424825, and GSM1518504 clustered together, while the remaining samples clustered separately. Subsequently, we performed WGCNA on the integrated dataset of TSE to screen for co-expression modules related to lipid metabolism subtypes (Figure 5C) and identified a total of 13 co-expressed gene modules. Finally, based on the expression patterns of the module genes and the grouping information of lipid metabolism subtypes, we assessed the correlation between gene modules and lipid metabolism subtypes (Figure 5D). We selected the gene module with the highest absolute correlation value (turquoise, *r* = −0.78, *p* = 0.0004) for subsequent analysis, which included 5,286 genes.

Further correlation analysis using a correlation scatterplot was conducted to assess the relationship between gene module membership and gene significance (Figure 5E), revealing a correlation of *r* = 0.76 and *p* < 1E-200. Module membership represented the relationship between genes and the module, while gene significance indicated the correlation between genes and phenotypic traits. Notably, genes highly significantly associated with a phenotype were often crucial elements within a module significantly associated with that phenotype.

Subsequently, we performed GO enrichment analysis based on the genes in the turquoise module (see Supplementary Table 4). The enriched GO functions primarily focused on lipid metabolism-related functions, such as phospholipid biosynthetic process, glycerophospholipid metabolic process, phospholipid metabolic process, glycerolipid metabolic process and others (Figure 5F).

### Risk model construction

**Figure 6:**
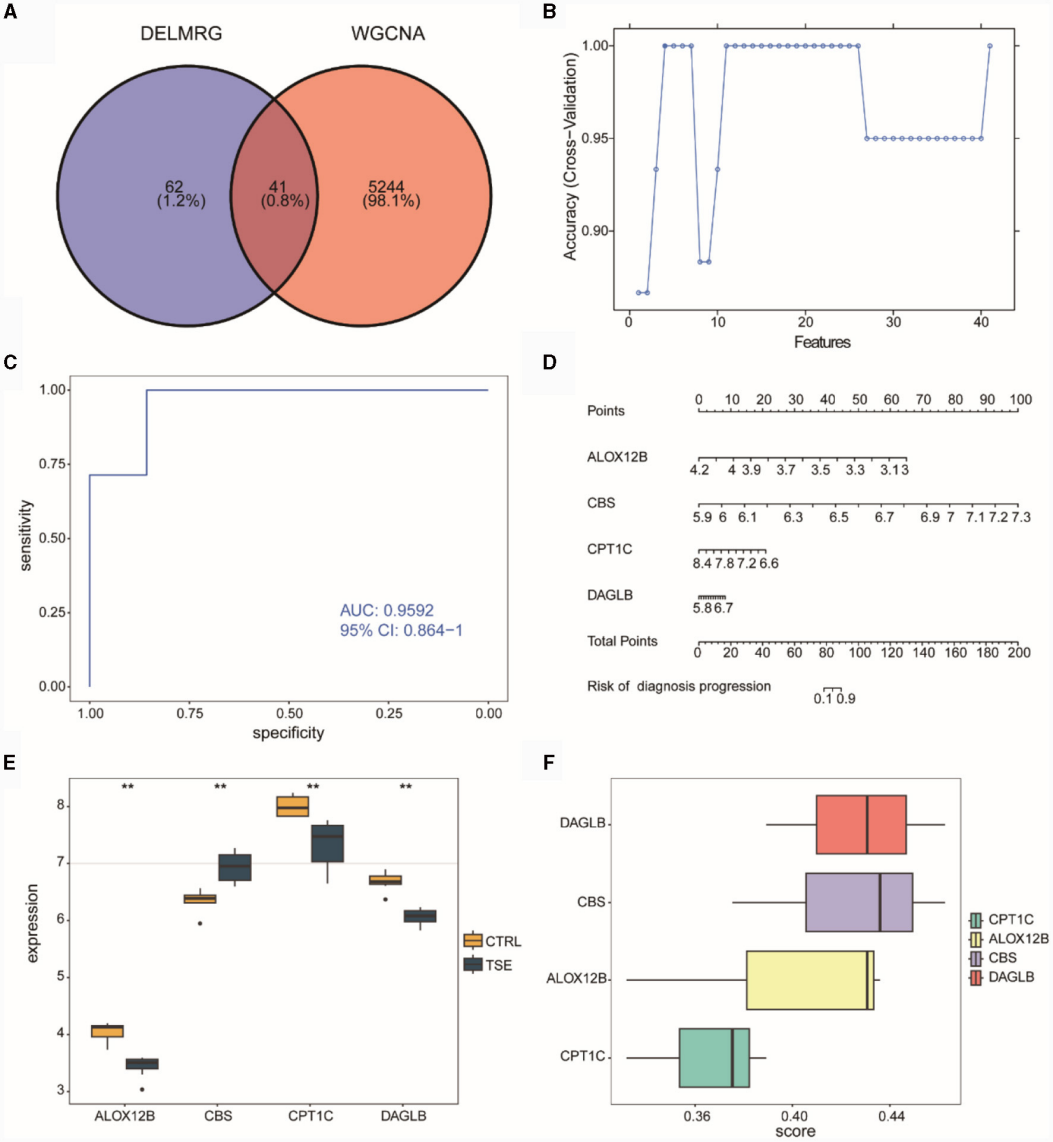
Construction of the diagnostic model in the integrated TSE dataset. **(A)** Venn diagram related to lipid metabolism subgroups in DELMRG and WGCNA. **(B)** Curve graph displaying the highest accuracy achieved through SVM-RFE feature selection. **(C)** ROC of the diagnostic model. **(D)** Nomogram for the diagnostic model. **(E)** Expression differences of the feature genes in the diagnostic model between the TSE group and the control group. **(F)** Ranking of the importance of feature genes in the diagnostic model through Friends analysis.

We intersected the WGCNA co-expressed module genes related to DELMRGs and TSE (Figure 6A), resulting in 41 differentially expressed genes (DEGs) that were associated with both lipid metabolism and TSE. Subsequently, we employed the SVM-RFE algorithm to select four feature genes from the 41 candidates, which could serve as diagnostic biomarkers for TSE disease grouping (Figure 6B). These four feature genes were ALOX12B, CBS, CPT1C, and DAGLB. We then used logistic regression to construct a risk diagnostic model for TSE disease grouping related to lipid metabolism, where the diagnosis score was calculated as follows: diagnosis score = (−64.748046) ^*^ expression (ALOX12B) + 99.234770 ^*^ expression (CBS) + (−22.507586) ^*^ expression (CPT1C) + 22.629042 ^*^ expression (DAGLB).

ROC curve analysis indicated that the constructed risk model exhibited high diagnostic accuracy for TSE, with an AUC value of 0.9592 (Figure 6C). Nomogram analysis was performed to assess the diagnostic capacity of the risk model, and a column chart (Nomogram) was generated (Figure 6D), which revealed that the genes ALOX12B and CBS made significant contributions to the diagnosis of TSE disease. Subsequently, we examined the expression differences of the four feature genes between the TSE group and the control group (Figure 6E). The results showed that the CBS gene had higher expression in the TSE group, while ALOX12B, CPT1C, and DAGLB had lower expression in the TSE group.

Furthermore, we conducted functional analysis (Figure 6F) to determine the importance of these four feature genes in GO functions. The analysis suggested that DAGLB and CBS played critical roles, indicating their potential significance as key genes.

### Immune infiltration analysis

**Figure 7:**
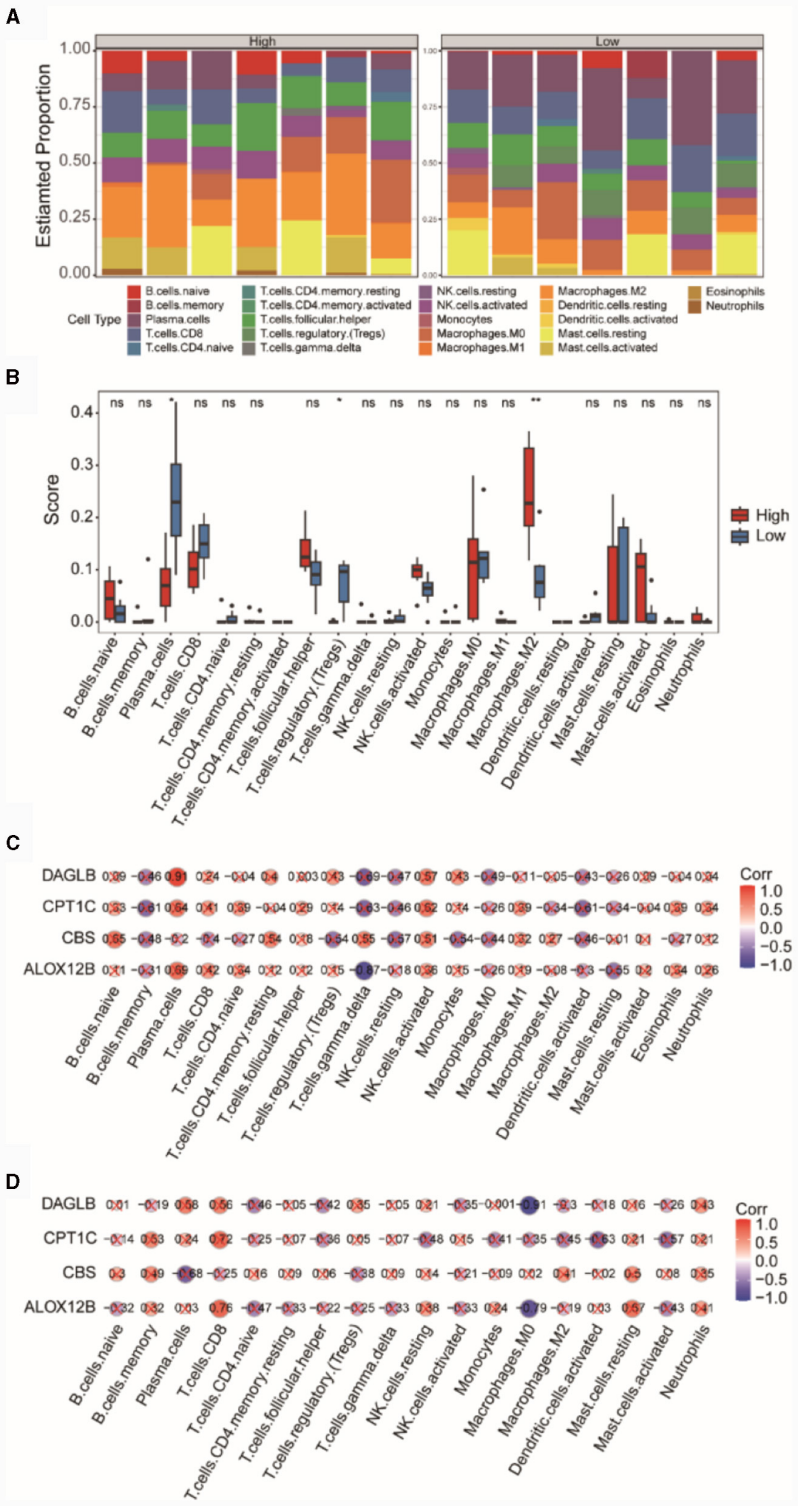
Analysis of immune cell infiltration in high and low diagnostic score groups. **(A)** Bar chart of immune cell compositions. **(B)** Box plot of immune cell infiltration. Pearson correlations between immune cells and the risk model genes within the high **(C)** and low **(D)** diagnostic score groups.

In the integrated TSE dataset, we computed the immune cell infiltration abundances of 22 different immune cell types in the high and low diagnostic score groups (Figure 7A). The results indicated a relatively balanced composition of immune cells in samples from the high and low diagnostic score groups. We separately compared the differences in the infiltration abundances of these 22 immune cell types between the high and low diagnostic score groups (Figure 7B). The results revealed that certain cells such as Plasma cells, T cells regulatory (Tregs), and Macrophages M2 showed significant differences (*p* < 0.05) between the high and low diagnostic score groups. Specifically, Macrophages M2 exhibited higher immune infiltration in the high diagnostic score group, while Plasma cells and T cells regulatory (Tregs) showed higher immune infiltration in the low diagnostic score group.

Subsequently, we presented the correlations between immune cells and the four diagnostic model genes in both the high and low diagnostic score groups. In the high diagnostic score group, DAGLB exhibited a significant positive correlation (*r* = 0.91, *p* = 0.0039) with Plasma cells (Figure 7C), whereas in the low diagnostic score group, DAGLB had the highest negative correlation (*r* = −0.91, *p* = 0.0049) with Macrophages M0 (Figure 7D).

### Differential functional analysis of high and low diagnostic score groups.

**Figure 8:**
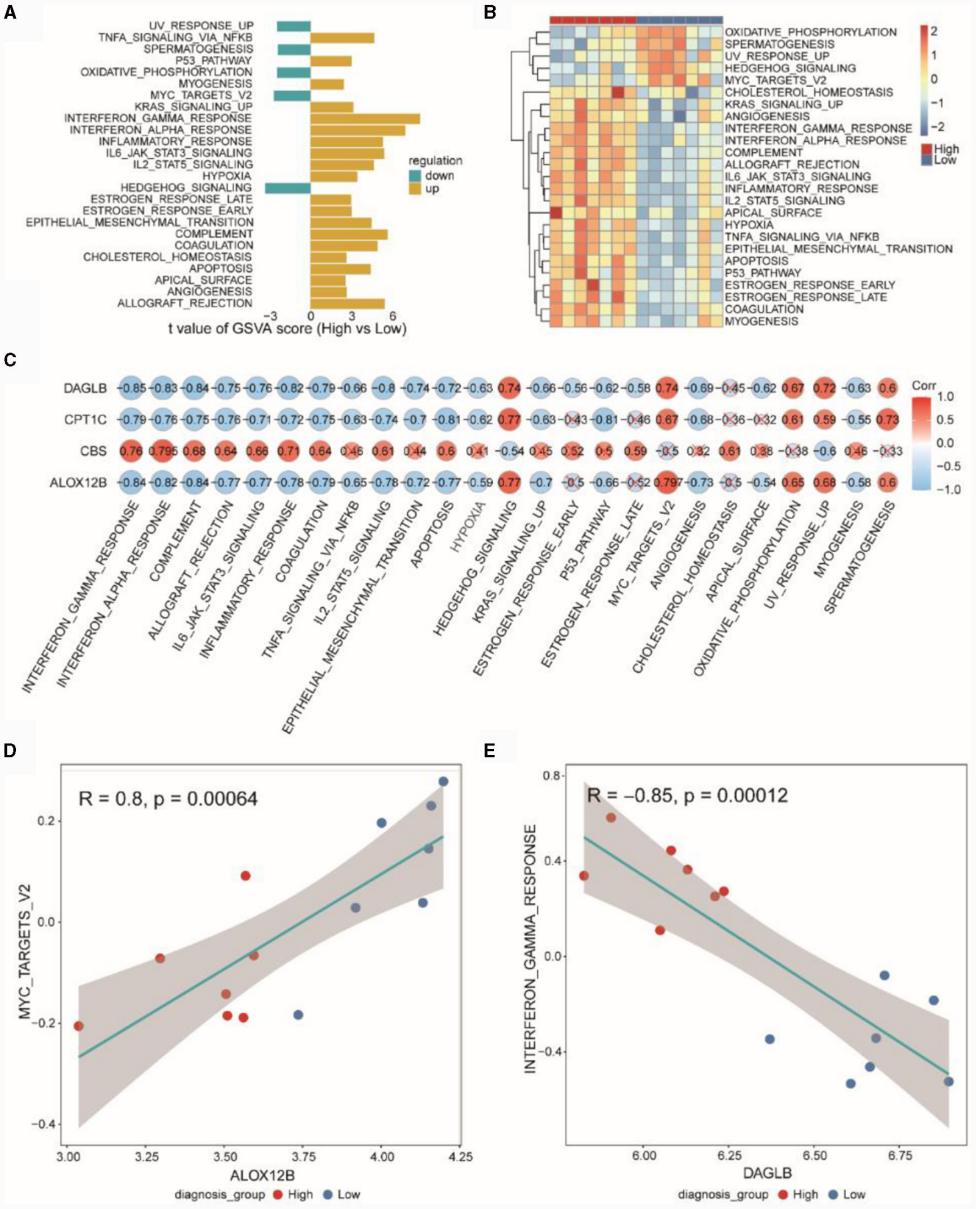
GSVA pathway analysis of high and low diagnostic score groups. **(A)** Differential HALLMARK pathways in high and low diagnostic score groups in the TSE integrated dataset. **(B)** Heatmap of GSVA scores for HALLMARK Pathways in the TSE integrated dataset. **(C)** Pearson correlation between GSVA Scores of HALLMARK pathways and expression of risk model genes. **(D)** Pearson correlation between the gene ALOX12B and the HALLMARK pathway MYCTARGETSV2. **(E)** Pearson correlation between the gene DAGLB and the HALLMARK pathway INTERFERONGAMMARESPONSE.

Based on the MSigDB HALLMARK gene set, we conducted GSVA. In Figure 8A, GSVA scores for pathways showed differences between the high and low diagnostic score groups (High vs. Low), with UVRESPONSEUP, SPERMATOGENESIS, OXIDATIVEPHOSPHORYLATION, MYCTARGETSV2 and HEDGEHOGSIGNALING exhibiting higher GSVA scores in the low diagnostic score group. Figure 8B presented the GSVA scores for differential HALLMARK pathways in the high and low diagnostic score groups. Figure 8C indicated the correlation between diagnostic genes and pathways, revealing that DAGLB, CPT1C, and ALOX12B were negatively correlated with most HALLMARK pathways, while CBS was positively correlated with most pathways. Figure 8D demonstrated a positive correlation (*r* = 0.8, *p* = 0.0006) between ALOX12B and MYCTARGETSV2, and Figure 8E showed a negative correlation (*r* = −0.85, *p* = 0.0001) between DAGLB and INTERFERONGAMMARESPONSE.

### Single cell data analysis

**Figure 9:**
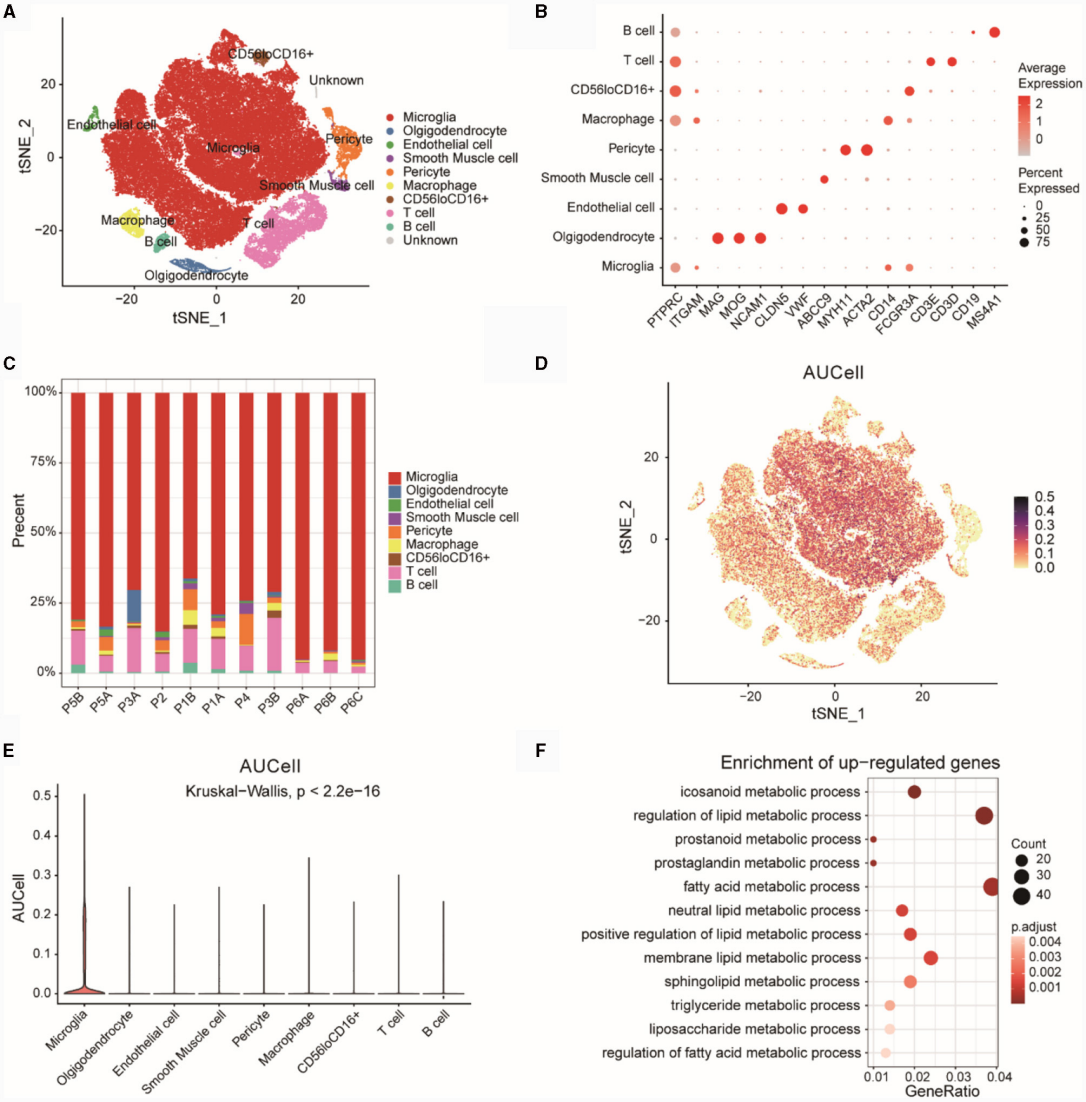
Analysis of GSE201048 single-cell data. **(A)** Cell type annotation for GSE201048 single-cell data, with each point representing a cell. **(B)** Bubble plot showing the expression of marker genes across various cell types, where deeper red indicates higher gene expression and larger points denote a higher proportion of cells expressing the gene within subpopulations. **(C)** Proportions of different cell types in the samples. **(D)** Scatterplot of AUCell scores for diagnostic model feature genes related to lipid metabolism across single-cell subtypes, with each point representing a cell and darker points indicating higher AUCell scores. **(E)** Violin plot depicting AUCell scores of diagnostic model feature genes related to lipid metabolism in single-cell subtypes, with the x-axis showing single-cell subtypes and the y-axis showing AUCell scores. **(F)** GO enrichment results for upregulated differentially expressed genes (DEGs) in Microglia compared to other cells, with the x-axis representing Gene ratio (the proportion of genes in the category out of all genes) and point size indicating the number of differentially expressed genes in the pathway, darker colors signify smaller *p*-adjusted values. GO, gene ontology.

We downloaded single cell data for epilepsy disease samples from PMID 35739273, which included 85,000 cells (without normal controls). The t-SNE plot revealed nine distinct cell subtypes after unsupervised clustering (Figure 9A). Figure 9B displayed the expression of marker genes for each cell type. The markers for various cell subtypes were derived from PMID 35739273. By examining the expression levels of CD45 (PTPRC), non-immune cells (CD45^low^) and immune cells (CD45^high^) could be distinguished. Cells with CD45^low^CD11b^low^ (CD11b was ITGAM) expression were identified as Microglia cells. Oligodendrocyte cells (CD45^low^CD56^high^) were marked by genes such as MAG, MOG, and NCAM1 (CD56), while Endothelial cell markers included CLDN5 and VWF. Smooth Muscle cell markers were ABCC9, and Pericyte cell markers were MYH11 and ACTA2. Among immune cells, CD11b^high^CD14^high^ cells were identified as Macrophages. T cell markers included CD3D and CD3E, B cell markers included MS4A1, and a subgroup of cells exhibited CD56^low^CD16^high^ expression (CD56 was NCAM1 and CD16 was FCGR3A).

Subsequently, we examined the proportions of cell subtypes in each sample (Figure 9C). The results showed that the distribution of various cell subtypes in the samples was relatively even, with no significant bias observed.

Using four risk model genes from the GSE201048 single-cell data, we scored each cell subtype with AUCell (Figure 9D) and visualized the results as box plots (Figure 9E). The findings indicated that Microglia subtypes had the highest AUCell scores, suggesting a strong association between Microglia cells and lipid metabolism functions.

Based on upregulated DEGs (Microglia vs. other cells), a GO analysis was conducted (Figure 9F; Supplementary Table 5). The results revealed significant enrichment of genes highly expressed in Microglia cells in pathways related to eicosanoid metabolic processes, regulation of lipid metabolic processes, prostanoid metabolic processes, and other LMRPs.

## Discussion

In our research, we identified a total of 103 DELMRGs. To further identify genes related to lipid metabolism for the construction of a diagnostic model for TSE, we took the intersection of the DELMRGs and the co-expression module genes related to TSE by WGCNA. This intersection yielded 41 genes that were both related to lipid metabolism and associated with TSE. We then used the SVM-RFE algorithm to select four feature genes that can serve as diagnostic biomarkers for the classification of TSE. These four feature genes are ALOX12B, CBS, CPT1C, and DAGLB.

Defects in ALOX12B, which subsequently lead to reduced epidermal LOX activity, result in the retention of scales in the stratum corneum of the epidermis. Disruption of the permeability barrier triggered by ALOX12B abnormalities due to an early stop mutation has been previously reported in a mouse model, where a complete lack of barrier formation was demonstrated, leading to rapid dehydration and death in the perinatal period (35).

CBS is a lytic enzyme that is mainly expressed in the liver. It is the rate-limiting enzyme in the transsulfuration pathway and is responsible for the metabolic conversion of homocysteine to the amino acid cysteine (36). CBS deficiency leads to hyperhomocysteinemia and impaired production of antioxidants such as hydrogen sulfide. Hepatic CBS plays an important role in the pathogenesis of NAFLD and in the defense against oxidative stress (37).

CPT1C is a member of the carnitine palmitoyltransferase 1 family and is involved in the regulation of physiological functions such as energy metabolism and feeding (38). CPT1C can have profound effects on brain physiology and total fatty acid profiles, which can be modulated by nutrients in the diet (39). Mice deficient in CPT1C exhibit behavioral and metabolic deficits. Overexpression of CPT1C in the brains of developmentally transgenic mice results in cerebellar hypoplasia. Thus, it is clear that CPT1C plays an important role in brain function (40).

Using DAGLB knockout mice, inactivation of DAGLB in mouse peritoneal macrophages reduced 2-AG, arachidonic acid and prostaglandins (41). A corresponding reduction in lipopolysaccharide-induced TNF-α release was also observed. These findings suggest a role for DAGLB in the lipid network that regulates the inflammatory response in macrophages (42).

The results of immune infiltration showed Macrophages.M2, higher immune infiltration in the high diagnostic score group and Plasma.cells, Tregs higher immune infiltration in the low diagnostic score group. Cerebral peripheral vascular macrophages are a special population of macrophages, and cerebral peripheral vascular macrophages are involved in the pathogenesis of neurodegenerative diseases, cerebrovascular dysfunction, autoimmune diseases, traumatic brain injury and epilepsy. They can act in a protective or deleterious manner on disease processes and stages (43). The number of Tregs in the brain was negatively correlated with seizure frequency in patients with epilepsy (44). Depletion of intracerebral Tregs promoted astrocytosis, microglia, inflammatory cytokine production, oxidative stress and neuronal loss in the hippocampus after status epilepticus seizures (45). Modulation of Tregs in epileptic brain tissue has therapeutic potential.

Microglia cells are highly correlated with lipid metabolic functions, and GO analysis showed that highly expressed genes in Microglia cells were significantly enriched in lipid metabolic pathways related to icosanoid metabolic process, regulation of lipid metabolic process, prostanoid metabolic process and so on. metabolic process, regulation of lipid metabolic process, prostanoid metabolic process and other lipid metabolic pathways. Myelin is required for the function of nerve axons in the central nervous system, and microglia are essential for maintaining myelin health. Oligodendrocyte status is associated with altered lipid metabolism (46).

Evidence accumulated over the past two decades has significantly bolstered the hypothesis that neuroinflammation plays a crucial role in epileptogenesis. This includes the activation of microglia and astrocytes, a cascade of inflammatory mediators being released, and the infiltration of peripheral immune cells from the bloodstream into the brain. Concurrently, an expanding corpus of preclinical studies indicates that anti-inflammatory agents, targeting key inflammatory components, demonstrate efficacy and hold promise in the treatment of epilepsy (47).

The pathophysiological consequences of microglial activation encompass exacerbated inflammation, modulation of neuronal activity, and the provocation of epileptic seizures (48). These studies collectively reinforce our belief in the significant role of microglia in TSE.

Furthermore, the mTOR signaling pathway is pivotal in neural development and neural circuit formation, primarily through the regulation of protein synthesis and autophagy. In the brain, inhibition of mTOR signaling diminishes the formation of autophagosomes, elevates lipopolysaccharide-induced proinflammatory cytokines in microglia, attenuates microglial activation, and mitigates astrocyte migration and proliferation, ultimately leading to a reduction in seizure severity (49).

We studied epilepsy due to tuberous sclerosis. But the last single-cell plot was validated with epilepsy due to cortical dysplasia. TSC and focal cortical dysplasia were focal malformations of cortical development highly associated with refractory epilepsy. TSC and FCD were mTOR disorders caused by a series of pathogenic variants in the target of rapamycin mechanism (mTOR) pathway genes leading to differential activation of mTOR signal (50). Considering that the electrical mechanisms of epilepsy are relatively similar, we extended the diagnostic genes to another type of epilepsy, and in this way did a single-cell analysis.

## Conclusion

Our research identified potential DELMRGs (ALOX12B, CBS, CPT1C, and DAGLB) in epilepsy, which may provide new ideas for studying the pathogenesis of Epilepsy. In the future, more experiments would be needed to further substantiate our conclusions.

## Data availability statement

The original contributions presented in the study are included in the article/Supplementary material, further inquiries can be directed to the corresponding authors.

## Ethics statement

Ethical approval was not required for the study involving humans in accordance with the local legislation and institutional requirements. Written informed consent to participate in this study was not required from the participants or the participants' legal guardians/next of kin in accordance with the national legislation and the institutional requirements.

## Author contributions

WW: Conceptualization, Data curation, Writing—review & editing. RY: Software, Writing—original draft. HW: Writing—original draft. ZX: Methodology, Writing—review & editing. YC: Writing—original draft. AW: Writing—original draft. XF: Conceptualization, Data curation, Writing—review & editing. WF: Conceptualization, Methodology, Writing—review & editing.
